# Central obesity, body mass index, metabolic syndrome and mortality in Mediterranean breast cancer patients

**DOI:** 10.1038/s41598-023-45439-y

**Published:** 2023-12-01

**Authors:** Anna Crispo, Livia S. A. Augustin, Assunta Luongo, Claudia Calderaio, Joao Breda, Sergio Coluccia, Alessandra Calabrese, Vittorio Marrazzo, Rosa Giannatiempo, Paola Trasacco, Elvira Palumbo, Sara Vitale, Giuseppe Porciello, Piergiacomo Di Gennaro, Roberta Caputo, Giuseppe Buono, Claudio Vernieri, Francesco Schettini, Maria Grimaldi, Flavia Nocerino, Egidio Celentano, Alfonso Amore, Mario Giuliano, Pietro De Placido, Carmine De Angelis, Roberto Bianco, Michelino De Laurentiis, Carlo La Vecchia, Grazia Arpino

**Affiliations:** 1 Epidemiology and Biostatistics Unit, Istituto Nazionale Tumori - IRCCS “Fondazione G. Pascale”, Naples, Italy; 2 Department of Clinical Medicine and Surgery, University of Naples Federico II, Naples, Campania Italy; 3 WHO Athens Quality of Care Office, Athens, Greece; 4 Department of Senology, Istituto Nazionale Tumori - IRCCS “Fondazione G. Pascale”, Naples, NA Italy; 5 Department of Pathology, Ospedale Evangelico Betania, Naples, Italy; 6 IFOM ETS, the AIRC Institute of Molecular Oncology, Milan, Italy; 7 Medical Oncology Department, Fondazione IRCCS Istituto Nazionale dei Tumori, Milan, Italy; 8 Translational Genomics and Targeted Therapies in Solid Tumors, IDIBAPS, Barcelona, Spain; 9 Medical Oncology Department, Hospital Clinic of Barcelona, Barcelona, Spain; 10 Faculty of Medicine and Health Sciences, Universitat de Barcelona, Barcelona, Spain; 11 Division of Surgery of Melanoma and Skin Cancer, Istituto Nazionale Tumori - IRCCS “Fondazione G. Pascale”, Naples, Italy; 12 Lester and Sue Smith Breast Center, Baylor College of Medicine, Houston, TX USA; 13 Department of Clinical Sciences and Community Health, Università degli Studi di Milano, Milan, Italy

**Keywords:** Cancer, Health care, Oncology, Risk factors

## Abstract

Obesity and metabolic disorders have been associated with poor outcomes in non-Mediterranean breast cancer (BC) patients. The purpose of this study was to investigate the prognostic potential of anthropometric variables in patients with early BC living in Southern Mediterranean region of Italy. We enrolled 955 consecutive early BC patients treated in hospitals in Naples between 2009 and 2013 (median follow-up 11.8-year ending 15/09/2022). Body mass index (BMI), waist circumference (WC), waist-to-hip ratio (WHR) and metabolic syndrome (MetS) were collected. All-cause and BC-specific mortality were calculated. At the last day of contact 208 (22%) patients had died, 131 (14%) from BC. High WC (≥ 88 cm) or WHR (> 0.85) and the MetS were significantly associated with moderately increased risk of all-cause mortality (HR=1.39, 1.62, 1.61, respectively). A significant increased risk of BC-specific mortality was found in obese patients, in those with high WC, high WHR and those with MetS (HR=1.72, 1.71, 1.80, 1.81, respectively). Central obesity significantly increased total and BC-specific mortality particularly in pre-menopausal women and in luminal subtypes, while in post-menopause MetS was a stronger risk factor. Obesity and MetS may impair the effectiveness of BC therapies hence active lifestyle interventions are encouraged.

## Introduction

Breast cancer (BC) is the first cause of cancer incidence in women and the fifth cause of cancer mortality globally^1^. In Europe, the highest incidence rates are observed in Northern and Western Europe and the lowest in Southern Europe. However, 5-year survival rates have been increasing in all European countries, particularly in Northern and Western Europe^2^. These differences in cancer incidence and survival could be related to several risk factors, among which non-modifiable and modifiable factors^2^. Among the latter, obesity has been associated with an increased risk of cancer and with poor outcomes in patients with cancer, including BC^3^.

In most studies, obesity is defined on the basis of body mass index (BMI) which has been used as a surrogate of total body adiposity. This approach is widely used in epidemiological studies as it can be simply calculated on the basis of participants' weight and height^4^. However, other anthropometric measurements such as waist circumference (WC), and waist-to-hip ratio (WHR) are used to estimate the presence of central adiposity and they are considered more accurate indicators of cancer risk than body weight^5,6^. In a study of American BC survivors, high WC and WHR were associated with worse overall and BC-specific survival^7^. However, American Black BC survivors may have different body composition and fat distribution compared to European Caucasians. They may also have different exposures to other modifiable risk factors such as food-related behaviors and the negative features of Western diets that are associated with an increased overall mortality among BC survivors^4,8^.

Herein we investigate the prognostic potential of the anthropometric variables BMI, WC and WHR together with a diagnosis of Metabolic Syndrome (MetS) and the presence of MetS components on clinical outcomes in women from Campania, a Southern Mediterranean region of Italy.

## Materials and methods

### Study population and design

A total of 955 BC patients were enrolled in this study between January 2009 and December 2013 at the Istituto Nazionale dei Tumori, “G. Pascale” and at the University Hospital “Federico II”, Naples, Italy. Anthropometric measurements (weight, height, waist and hip circumference), clinical data (age, menopausal status, type of adjuvant therapy, MetS components) and tumor characteristics were reported at the enrollment, before starting systemic (neo) adjuvant therapy. Median (min, max) time of follow-up calculated up to June 15, 2022 was 11.8 years (8.9, 14.5). The follow-up was performed via telephone surveys in which operators collected data on vital health status. A detailed description of the study population and design can be found in an earlier study^9^.

The study was approved by the Institutional Review Board of the University of Naples Federico II (IRB approval number 75/15) and participants provided written informed consent to participate. The patients’ records and data were anonymized and de-identified prior to analysis. The study was conducted in accordance with legal and regulatory requirements, as well as the general principles set forth in the International Ethical Guidelines for Biomedical Research Involving Human Subjects (Council for International Organizations of Medical Sciences 2002), Guidelines for GCP (ICH 1996) and addendum, and the Declaration of Helsinki (World Medical Association 1996 and its amendments). In addition, the study was conducted in accordance with the protocol and applicable local regulatory requirements and laws.

### Anthropometry

BMI information was available for 933 patients and categorized according to canonical BMI ranges^10^. Hip circumference and WC measurements (in cm) were collected from 901 and 900 patients, respectively. WC was also categorized according to NCEP-ATP III criteria (<88; ≥88 cm)^11^. WHR was calculated as the ratio between waist and hip circumferences, and categorized as ≤ 0.85 or > 0.85^12^.

### Metabolic syndrome and its components

MetS was defined according to NCEP-ATP III criteria^11^. Complete data to assess MetS were available for 718 patients (75%), and for 626 of them (66%) we were able to collect information about the specific number of MetS components (0, 1–2, ≥ 3), while for the remaining 92 patients (10%) we did not have sufficient information to attribute a score of MetS component of 0, 1 or 2 (Table 1).

**Table 1:** Patients and tumor characteristics, all-cause and BC-specific deaths, overall and by menopausal-status. Naples, Italy, 2009-2022.

Variables	All			Pre-menopausal			Post-menopausal		
	N^1^ (%)	Deaths from all-causes	Deaths from breast cancer	N^1^ (%)	Deaths from all-causes	Deaths from breast cancer	N^1^ ﻿(%)	Deaths from all-causes	Deaths from breast cancer
	955	208	131	369	34	31	586	174	100
Center									
IRCCS G. Pascale	526 (55)	137	87	196 (53)	20	19	330 (56)	117	68
Policlinico Federico II	429 (45)	71	44	173 (47)	14	12	256 (44)	57	32
Age (years)									
< 40	93 (10)	15	14	92 (25)	15	14	1 (0)	0	0
40–49	249 (26)	21	17	232 (63)	16	14	17 (3)	5	3
50–59	257 (27)	52	41	45 (12)	3	3	212 (36)	49	38
≥ 60	356 (37)	120	59	0	0	0	356 (61)	120	59
ER									
Negative (0)	172 (18)	49	36	57 (15)	10	9	115 (20)	39	27
Positive (>0)	781 (82)	159	95	311 (84)	24	22	470 (80)	135	73
PGR									
Negative (0)	217 (23)	65	47	68 (18)	13	12	149 (25)	52	35
Positive (>0)	736 (77)	143	84	300 (81)	21	19	436 (74)	122	65
Ki67>20									
Negative (<20%)	547 (58)	95	53	172 (47)	11	11	295 (50)	84	42
Positive (≥20%)	398 (42)	112	77	194 (53)	22	19	287 (49)	90	58
Surrogate molecular subtypes									
Luminal A-like	310 (33)	59	30	110 (30)	3	3	200 (34)	56	27
Luminal B-like/HER2-	341 (37)	75	50	141 (38)	15	13	200 (34)	60	37
HER2+	152 (16)	34	23	67 (18)	6	6	85 (15)	28	17
Triple negative	123 (13)	33	26	40 (11)	9	8	83 (14)	24	18
HR status									
HR–	158 (17)	48	36	49 (13)	9	9	109 (19)	39	27
HR+	795 (83)	160	95	319 (86)	25	22	476 (81)	135	73
Cancer stage									
I–IIA	614 (64)	110	57	244 (66)	18	16	370 (63)	92	41
IIB	125 (13)	30	19	46 (13)	7	7	79 (14)	23	12
IIIA–IIIC	174 (18)	56	45	68 (18)	8	7	106 (18)	48	38
Tumor dimension (T)									
T1	530 (56)	96	54	215 (58)	15	13	315 (54)	81	41
T2	352 (37)	89	60	125 (34)	16	15	227 (39)	73	45
T3–T4	49 (5)	17	13	22 (6)	3	3	27 (5)	14	10
Axillary nodal status (N)									
N0	513 (54)	92	42	193 (52)	15	13	320 (55)	77	29
N+	413 (43)	107	82	169 (46)	18	17	244 (42)	89	65
Histological grade									
G1	57 (6)	7	1	26 (7)	0	0	31 (5)	7	1
G2	386 (40)	85	48	140 (38)	10	10	246 (42)	75	38
G3	490 (51)	105	73	197 (53)	21	18	293 (50)	84	55
Cancer type									
Invasive ductal carcinoma	710 (74)	158	106	281 (76)	31	28	429 (73)	127	78
Invasive lobular carcinoma	149 (16)	32	16	52 (14)	1	1	97 (17)	31	15
Tubular carcinoma	31 (3)	4	2	15 (4)	0	0	16 (3)	4	2
Other	65 (7)	14	7	21 (6)	2	2	44 (8)	12	5
Treatments									
No therapy	59 (7)	7	3	19 (5)	2	1	40 (7)	5	2
Adjuvant/neoadjuvant	120 (14)	31	22	50 (14)	9	9	70 (12)	22	13
Hormone	678 (79)	118	65	266 (72)	13	12	412 (70)	105	53
Body mass index, kg/m^2^									
N	933	202	127	359 (97)	33	30	574 (98)	169	97
< 25	341 (37)	57	36	194 (53)	15	12	147 (25)	42	24
25–30	317 (34)	67	36	112 (30)	12	12	205 (35)	55	24
≥ 30	275 (29)	78	55	53 (14)	6	6	222 (38)	72	49
Waist circumference, cm									
N	900	192	124	347 (94)	31	29	553 (94)	162	95
< ﻿88	410 (46)	64	41	225 (61)	15	14	185 (32)	49	27
≥ 88	490 (54)	129	83	122 (33)	16	15	368 (63)	113	68
Waist-to-hip ratio, u									
N	899	192	124	348 (94)	31	29	551 (94)	161	95
≤ 0.85	322 (36)	46	32	184 (50)	13	12	138 (24)	33	20
> 0.85	577 (64)	146	92	164 (44)	18	17	413 (71)	128	75
Metabolic syndrome (MetS)^2^									
No	545 (76)	95	65	271 (93)	24	23	274 (64)	71	42
Yes	173 (24)	64	42	21 (7)	2	2	152 (36)	62	40
MetS components									
None	122 (19)	11	10	81 (36)	6	6	41 (10)	5	4
1–2	331 (53)	75	48	125 (55)	15	14	206 (52)	60	34
≥ 3	173 (28)	64	42	21 (69)	2	2	152 (38)	62	40

*ER* estrogen receptor, *PGR* progesterone receptor, *HER2* human epidermal growth factor receptor-2, *HR* hormone receptor.

^1^For some variables the sum does not add up to the total due to missing values.

^2^MetS was defined by the presence of 3 to 5 of the following criteria: WC ≥88 cm, blood pressure ≥ 130/ ≥ 85 mmHg, fasting (at least 8-hour fasting) concentration of serum triglycerides ≥ 150 mg/dL, high-density protein cholesterol (HDL-C) < 50 mg/dL and fasting plasma glucose concentration ≥ 110 mg/dL.

### Tumor characteristics

Immunohistochemical (IHC)-based surrogates of molecular BC subtypes were assigned based on the criteria established by the 13th St Gallen International Breast Cancer Conference (2013) Expert Panel^13,14^.

### Statistical analyses

Survival time was calculated from the date of BC diagnosis to the date of patient death or to the end of the follow-up period (June 15th 2022), which ever occurred first. The calculation of all-cause and BC-specific mortality in patients lost to follow-up was censored on the last day in which the patient was considered free from the event.

The corresponding adjusted hazard ratios (HRs) and 95% confidence intervals (CIs) were calculated using adjusted Cox multivariable proportional hazards regression models, and a stepwise approach if necessary. Adjustment variables included terms for age (≤40, 41–60, >60), center, tumor stage (I–IIA; IIB; IIIA–IIIC) and molecular subtypes (HR+/HER2–, HER2+, TN). The HRs were calculated for BMI, WC and WHR as categorical variables; moreover, the HRs for an increase of 5 units (U) (kg/m^2^) of BMI, 10-U (cm) of WC and 0.1-U of WHR were also estimated when these variables were evaluated as continuous ones in the models. A stratified analysis was also performed by molecular subtypes and by luminal status to investigate the association between anthropometric and metabolic measurements and all-cause or BC-specific mortality. All statistical analyses were performed using R version 4.1.3.

## Results

This study enrolled 955 women with early BC. Mean age 55.3±12.5 years, and 61% of patients were post-menopausal. Of 955 patients enrolled, 208 patients died from any cause (34 in pre- and 174 in post-menopausal status), and of these 131 died from BC (31 in pre- and 100 in post-menopausal status). BC-specific death was not available for 80 patients but were included in the overall mortality count, while 3 patients were lost to follow-up during the course of the study, therefore no information about their vital status was available. The characteristics of patients and their tumors, as well as the number of patients undergoing death events, are summarized in Table 1. Regarding BC subtypes, 33% and 37% of patients had Luminal A-like and Luminal B-like BC, respectively, 16% of patients had HER2+ BC (either HR+ or HR–), and 13% of patients had triple-negative BC (TNBC). Overall, 83% of all patients had HR+ tumors. Two-thirds (64%) had stage I–IIA disease. The most frequent histological tumor grades were G2 and G3 (40% and 51%, respectively). Invasive ductal carcinoma (IDC) was the main histological type (74%). Regarding pharmacologic treatments, most patients (79%) received endocrine therapy, while 14% received (neo) adjuvant chemotherapy (CT), thus reflecting a population of patients with relatively low clinical risk of tumor recurrence. Similar distributions of tumor characteristics were observed in pre-and post-menopausal women.

Obesity (BMI≥30 kg/m^2^) was found in 29% of the whole study cohort, 14% in pre- and 38% in post-menopausal women. Approximately 24% of patients met the criteria for a diagnosis of MetS, 7% in pre- and 36% in post-menopause, while the presence of 1-2 criteria was found in 53% of patients overall, 55% in pre- and 52% in post-menopause.

All-cause and BC-specific mortality were 78% and 85%, respectively (Additional Fig. 1). Table 2 summarizes anthropometric/metabolic variables and their association with all-cause or BC-specific mortality, overall or according to menopausal status. Although obese patients had a higher risk of death compared to normal weight/overweight patients (Additional Fig. 2), multivariable analysis did not show an independent association between BMI, as evaluated as a categorical variable, and all-cause mortality. However, each 5.0-U increase in BMI was associated with an increased risk of all-cause mortality (HR=1.17, 95% CI 1.02–1.34, p = 0.030). Unlike BMI, a high WC and WHR were associated with a moderately increased risk of all-cause mortality also when evaluated as dichotomous variables (WC ≥ 88 cm, HR = 1.39, 95% CI 1.00–1.94; WHR > 0.85, HR = 1.62, 95% CI 1.12–2.37), and this association retained statistical significance when WC and WHR were evaluated as continuous variables (HRs = 1.16, 95% CI 1.05–1.29 and HR = 1.27, 95% CI 1.07–1.50 respectively). Lastly, we found an association between MetS components and the risk of all-cause mortality (HR = 1.61, 95% CI 1.12–2.32). In particular, patients with ≥3 MetS components had almost quadrupled the risk of death versus patients without MetS (HR = 3.94, 95% CI 1.88–8.26).

**Table 2:** Association of anthropometric measures and MetS with all-cause and BC-specific mortality, overall and by menopausal status, Naples, Italy, 2009-2022.

	All				Pre-menopausal				Post-menopausal			
	Deaths from all-causes		Deaths from breast cancer		Deaths from all-causes		Deaths from breast cancer		Deaths from all-causes		Deaths from breast cancer	
	HR* (95% CI)	p**	HR* (95% CI)	p**	HR* (95% CI)	p**	HR (95% CI)	p**	HR* (95% CI)	p**	HR (95% CI)	p**
Body mass index, kg/m^2^		0.345		**0.029**		0.291		0.090		0.277		**0.032**
< 25	1		1		1		1		1		1	
25–30	0.99 (0.67-1.47)		1.03 (0.62-1.73)		1.79 (0.77-4.13)		**2.44 (1.00-5.95)**		0.73 (0.48-1.12)		**0.52 (0.28-0.98)**	
≥ 30	1.25 (0.85-1.84)		**1.72 (1.06–2.78)**		1.91 (0.70–5.26)		2.60 (0.90–7.48)		0.95 (0.63–1.41)		1.06 (0.62–1.79)	
Per 5 U	**1.17 (1.02–1.34)**	**0.030**	**1.31 (1.11–1.55)**	**0.002**	**1.43 (1.04–1.96)**	**0.028**	**1.58 (1.15–2.18)**	**0.005**	1.06 (0.91–1.23)	0.457	1.15 (0.94–1.41)	0.189
Waist circumference, cm		**0.053**		**0.014**		**0.007**		**0.006**		0.926		0.536
< 88	1		1		1		1		1		1	
≥ 88	**1.39 (1.00–1.94)**		**1.71 (1.12–2.61)**		**2.94 (1.35–6.42)**		**3.09 (1.37–6.94)**		1.02 (0.71–1.46)		1.17 (0.72–1.89)	
Per 10 U	**1.16 (1.05–1.29)**	**0.005**	**1.24 (1.10–1.40)**	**0.001**	**1.33 (1.01–1.76)**	**0.046**	**1.39 (1.05–1.86)**	**0.023**	1.09 (0.97–1.23)	0.141	1.15 (0.99–1.34)	0.065
Waist-to-hip ratio, u		**0.011**		**0.014**		**0.036**		**0.035**		0.367		0.395
≤ 0.85	1		1		1		1		1		1	
> 0.85	**1.62 (1.12–2.37)**		**1.80 (1.13–2.86)**		**2.38 (1.06–5.33)**		**2.46 (1.06–5.71)**		1.21 (0.80–1.84)		1.27 (0.73–2.20)	
Per 0.1 U	**1.27 (1.07–1.50)**	**0.005**	**1.33 (1.08–1.63)**	**0.007**	1.54 (0.91–2.60)	0.105	1.61 (0.93–2.77)	0.089	1.12 (0.92–1.36)	0.246	1.15 (0.90–1.47)	0.254
Metabolic syndrome (MetS)		**0.010**		**0.010**		0.463		0.476		0.193		0.103
No	1		1		1		1		1		1	
Yes	**1.61 (1.12–2.32)**		**1.81 (1.51–2.85)**		1.79 (0.38–8.47)		1.76 (0.37–8.38)		1.29 (0.88–1.89)		1.50 (0.92–2.45)	
MetS components		**0.001**		**0.008**		0.170		0.218		0.099		0.150
None	1		1		1		1		1		1	
1–2	**2.92 (1.44–5.91)**		**2.45 (1.15–5.25)**		2.99 (0.95–9.44)		2.79 (0.87–8.91)		2.39 (0.95–6.01)		2.09 (0.73–5.95)	
≥ 3	**3.94 (1.88–8.26)**		**3.60 (1.60–8.11)**		2.76 (0.45–16.89)		2.55 (0.42–15.63)		**2.77 (1.09–7.06)**		2.73 (0.95–7.84)	

*Cox proportional hazard ratio (HR) adjusted by terms of: age (≤ 40, 41–60, > 60), center (IRCCS G. Pascale, Policlinico Federico II), cancer stage (I–IIA, IIB, IIIA–IIIC) and molecular subtypes (Luminal A, Luminal B, HER 2+, TN); **For the entire variable the p-value refers to Wald Test, for numerical variable z-Test p-value was reported.

Significant results are shown in bold.

Regarding BC-specific mortality risk, it was higher in obese patients (BMI ≥ 30 kg/m^2^, HR = 1.72, 95% CI 1.06–2.78) and for each 5.0-U increase in BMI (HR = 1.31, 95% CI 1.11–1.55). In addition, patients with a WC ≥ 88 cm had a 71% increased risk of BC-specific mortality (HR = 1.71, 95% CI 1.12–2.61). These results were confirmed for each 10-U increase in WC (HR = 1.24, 95% CI 1.10–1.40). We also found an independent association between higher WHR and an increased risk of BC-specific mortality, both when WHR was evaluated as a categorical variable (for WHR > 0.85, HR = 1.80, 95% CI 1.13–2.86) and when it was considered as a continuous variable (for each 0.1-U increase in WHR, HR = 1.33, 95% CI 1.08–1.63). The presence of MetS was associated with an 81% increased risk of BC-specific mortality (HR = 1.81, 95% CI 1.51–2.85). In addition, the presence of 1–2 or ≥3 MetS components was associated with significantly higher risk of BC-specific mortality (HR = 2.45, 95% CI 1.15–5.25 and HR = 3.60, 95% CI 1.60–8.11, respectively).

Among pre-menopausal patients, a 5-U increase in BMI was associated with an increased risk for all-cause or BC-specific mortality (HR = 1.43, 95% CI 1.04–1.96 and HR = 1.58, 95% CI 1.15–2.18, respectively). A high WC was independently associated with an increased risk of all-cause and BC-specific mortality both as a categorical variable (WC > 88, HR = 2.94, 95% CI 1.35–6.42 and HR = 3.09, 95% CI 1.37–6.94, respectively) and as a continuous variable (HR = 1.33, 95% CI 1.01–1.76 and HR= 1.39, 95% CI 1.05–1.86 respectively). Similarly, BC patients with WHR > 0.85 had a 2-fold increased risk of all-cause and BC-specific mortality (HR = 2.38, 95% CI 1.06–5.33 and HR = 2.46, 95% CI 1.06–5.71 respectively). Among post-menopausal women we only found an increased risk of all-cause mortality in the presence of ≥ 3 MetS components (HR = 2.77, 95% CI 1.09–7.06).

Then, we moved to study the prognostic impact of anthropometric and metabolic variables according to tumor biology. Table 3 shows the results of multivariable models according to surrogate molecular subtypes. In patients with HR+/HER2- disease, we found a slightly increased risk in all-cause mortality and BC-related death for every 5-U increase in BMI (HR = 1.21, 95% CI 1.01–1.44 and HR = 1.58, 95% CI 1.11–1.72, respectively). We also found a borderline significant increase in BC-specific mortality risk in patients with WC≥88 cm (HR = 1.75, 95% CI 0.99–3.06), as well as a statistically significantly increased risk in either all-cause or BC-specific mortality for each 10-U increase in WC (HR = 1.19, 95% CI 1.06–1.34 and HR = 1.28, 95% CI 1.11–1.48, respectively). Patients with high WHR also had higher risk of all-cause mortality, both when WHR was considered as a dichotomous (WHR > 0.85, HR = 1.85, 95% CI 1.14–2.99) and as a continuous variable (HR = 1.26, 95% CI 1.04–1.53). Moreover, an increased risk of death (all-cause and BC-specific) was observed for each 0.1-U increase in WHR (HR = 1.26, 95% CI 1.04–1.53 and HR=1.32, 95% CI 1.03–1.68, respectively). Finally, we found significantly increased risk of all-cause or BC-specific mortality in patients with 1-2 MetS components (HR = 3.86, 95% CI 1.37–10.84 and HR = 3.68, 95% CI 1.11–12.22, respectively) and ≥ 3 MetS components (HR = 4.65, 95% CI 1.59–13.57 and HR = 4.62, 95% CI 1.30–16.46, respectively).

**Table 3:** Association of anthropometric measures and MetS with all-cause and BC-specific mortality by molecular subtypes, Naples, Italy, 2009–2022.

	HR+				HER 2+				TN			
	Deaths from all-causes		Deaths from breast cancer		Deaths from all-causes		Deaths from breast cancer		Deaths from all-causes		Deaths from breast cancer	
	HR* (95% CI)	p**	HR* (95% CI)	p**	HR* (95% CI)	p**	HR* (95% CI)	p**	HR* (95% CI)	p**	HR* (95% CI)	p**
Body mass index, kg/m^2^		0.433		0.087		0.501		0.183		0.835		0.855
< 25	1		1		1		1		1		1	
25–30	1.10 (0.68–1.78)		1.03 (0.53–1.99)		0.93 (0.36–2.42)		1.03 (0.29–3.61)		0.76 (0.26–2.17)		1.25 (0.38 –4.04)	
≥ 30	1.35 (0.82–2.21)		1.78 (0.94–3.37)		1.53 (0.64–3.68)		2.47 (0.84–7.27)		0.77 (0.28–2.15)		0.88 (0.25–3.12)	
Per 5 U	**1.21 (1.01–1.44)**	**0.035**	**1.58 (1.11–1.72)**	**0.004**	1.23 (0.89–1.68)	0.207	1.42 (0.94–2.16)	0.096	1.11 (0.75–1.14)	0.616	1.16 (0.74–1.84)	0.519
Waist circumference, cm		0.088		0.052		0.245		0.087		0.999		0.993
< 88	1		1		1		1		1		1	
≥ 88	1.44 (0.95–2.18)		1.75 (0.99–3.06)		1.59 (0.73–3.49)		2.30 (0.89–5.99)		1.00 (0.42–2.41)		0.99 (0.37–2.68)	
Per 10 U	**1.19 (1.06–1.34)**	**0.004**	**1.28 (1.11–1.48)**	**0.001**	**1.36 (1.04–2.77)**	**0.024**	**1.62 (1.10–2.37)**	**0.014**	0.94 (0.70–1.27)	0.696	0.94 (0.70–1.34)	0.849
Waist-to-hip ratio		**0.013**		0.087		0.735		0.176		0.357		0.231
≤ 0.85	1		1		1		1		1		1	
> 0.85	**1.85 (1.14–2.99)**		1.67 (0.93–3.00)		1.14 (0.53–2.44)		1.97 (0.74–5.27)		1.75 (0.53–5.78)		2.27 (0.59–8.71)	
Per 0.1 U	**1.26 (1.04–1.53)**	**0.019**	**1.32 (1.03–1.68)**	**0.028**	1.42 (0.91–2.21)	0.124	1.49 (0.86–2.59)	0.154	1.30 (0.69–2.42)	0.417	1.32 (0.67–2.61)	0.418
Metabolic syndrome (MetS)		0.125		0.169		**0.005**		**0.002**		0.766		0.903
No	1		1		1		1		1		1	
Yes	1.42 (0.91–2.23)		1.52 (0.84–2.76)		**3.45 (1.45–8.22**)		**5.05 (1.80–14.20)**		1.18 (0.41–3.44)		1.08 (0.33–3.51)	
MetS components		**0.019**		0.061		**0.034**		**0.016**		0.198		0.245
None	1		1		1		1		1		1	
1–2	**3.86 (1.37–10.84)**		**3.68 (1.11–12.22)**		0.96 (0.29–3.21)		0.55 (0.14–2.14)		6.99 (0.84–58.08)		6.15 (0.73–52.10)	
≥ 3	**4.65 (1.59–13.57)**		**4.62 (1.30–16.46)**		3.03 (0.83–11.03)		2.94 (0.75–11.56)		5.69 (0.59–54.77)		4.72 (0.47–47.87)	

*HR+*, hormone receptor, *HER2+* human epidermal growth factor receptor-2, *TN* triple negative.

^*^Cox proportional hazard ratio (HR) adjusted by terms of: age (≤ 40, 41–60, > 60), center (IRCCS G. Pascale, Policlinico Federico II), cancer stage (I–IIA, IIB, IIIA–IIIC);

^**^For the entire variable the p-value refers to Wald Test, for numerical variable z-Test p-value was reported.

Significant results are shown in bold.

In patients with HER2+ BC, each 10-U increase of WC was associated with an increased risk of all-cause or BC-specific mortality (HR = 1.36, 95% CI 1.04–2.77; HR = 1.62, 95% CI 1.10–2.37, respectively). HER2+ BC patients meeting the criteria of a MetS diagnosis also had an increased risk of all-cause and BC-specific mortality (HR = 3.45 95% CI 1.45–8.22 and HR = 5.05, 95% CI 1.80–14.20, respectively). Similarly, the presence of at least 3 components of MetS was associated with a trend towards increased mortality (all-cause p = 0.005 and BC-specific p = 0.002).

**Figure 1:**
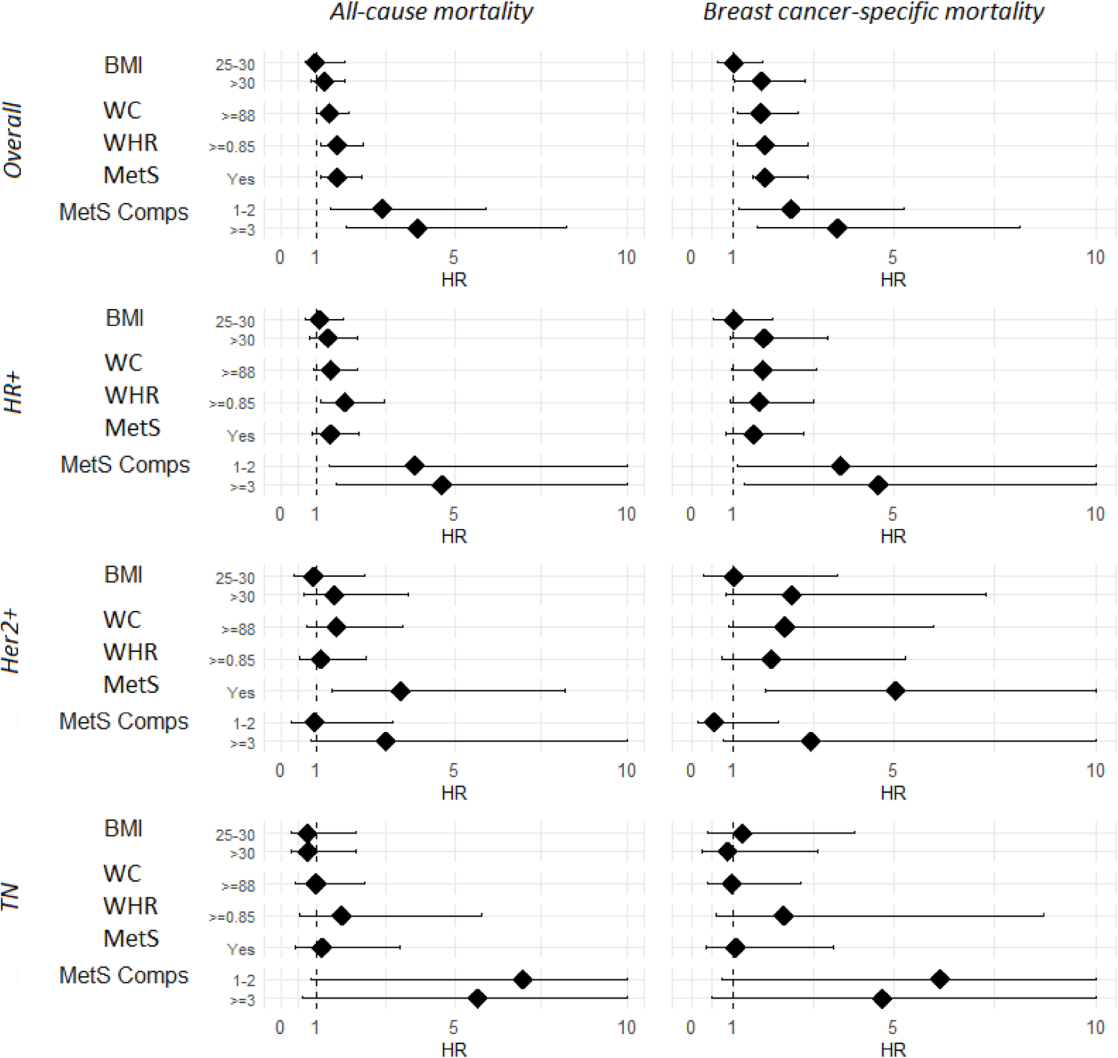
Forest plot for BMI, WC, WHR, MetS, MetS components of all-cause and BC-specific mortality. Forest plot of the HRs and 95% CI of All-cause and BC-specific mortality for BMI, WC, WHR, MetS, MetS components and molecular subtypes: overall and by molecular subtypes. *HR*+ hormone receptor positive, *HER2*+ human epidermal growth factor receptor-2 positive, *TN* triple negative, *BMI* body mass index (BMI is calculated as weight in kilograms divided by height in meters squared), *WC* waist circumference (in centimeters), *WHR* waist-to-hip ratio (WHR calculated as the ratio between waist and hip circumferences), *MetS* metabolic syndrome (defined according to NCEP-ATP III criteria), *MetS comps* metabolic syndrome components (1-2, ≥ 3).

Lastly, in patients with TNBC we did not find an independent association between BMI, WC, WHR, or MetS categories, and all-cause and BC-specific mortality (Table 3 and Fig. 1).

Luminal BC is a highly heterogeneous group of diseases, which includes more and less clinically aggressive forms, such as Luminal B-like and Luminal A-like patients. For this reason, among HR+/HER2- BC patients we separately evaluated the association between anthropometric/metabolic variables and all-cause or BC-specific mortality in patients with Luminal A-like and Luminal B-like disease (Table 4). BMI and WC were not associated with either all-cause or BC-specific mortality in Luminal A-like patients. However, an increased risk in BC-specific mortality for each 5-U increase in BMI (HR = 1.43, 95% CI 1.02–2.00) was found. When WC was considered as a continuous variable there was an increased risk for each 10-U increase of all-cause and BC-specific mortality (HR = 1.28, 95% CI 1.05–1.55 and HR = 1.41, 95% CI 1.07–1.86, respectively). Similarly, high WHR was associated with an increased risk of all-cause and BC-specific mortality for each 0.1-U increase in WHR (HR = 1.74, 95% CI 1.28–2.39 and HR = 1.92, 95% CI 1.27–2.90, respectively). The presence of MetS was associated with an increased risk of all-cause and BC-specific mortality (HR = 2.84, 95% CI 1.47–5.48 and HR = 2.81, 95% CI 1.15–6.86, respectively). In addition, the presence of ≥ 3 MetS components was associated with a significantly higher risk of all-cause and BC-specific mortality (HR = 2.98, 95% CI 1.52–5.88 and HR = 2.80, 95% CI 1.14–6.88, respectively). In Luminal B-like BC patients there was a significantly higher risk of mortality (all-cause and BC-specific) for each 10-U increase in WC (HR=1.18, 95% CI 1.01–1.38 and HR=1.22, 95% CI 1.01–1.46, respectively). Regarding MetS, the presence of 1-2 MetS components was associated with a significantly higher risk of all-cause mortality compared with no MetS components (HR = 3.07, 95% CI 1.0–8.81) (Table 4).

**Table 4:** Association of anthropometric or MetS variables and all-cause or BC-specific mortality by luminal subtypes (A vs. B).

Variable	Luminal A					Luminal B				
	Deaths/N total cases	Deaths from all-causes		Deaths from breast cancer		Deaths/N total cases	Deaths from all-causes		Deaths from breast cancer	
		HR* (95% CI)	p**	HR (95% CI)	p**		HR* (95% CI)	p**	HR (95% CI)	p**
Body mass index, kg/m^2^			0.247		0.090			0.675		0.386
< 25	11/103	1		1		19/117	1		1	
25–30	21/110	1.28 (0.61-2.69)		0.75 (0.23–2.49)		29/127	0.98 (0.52–1.87)		1.05 (0.47–2.36)	
≥ 30	27/91	1.79 (0.87–3.67)		2.05 (0.78–5.36)		26/92	1.24 (0.64–2.40)		1.59 (0.71–3.56)	
Per 5.0 U	59/304	1.24 (0.97–1.58)	0.084	**1.43 (1.02–2.00)**	**0.038**	74/336	1.18 (0.93–1.49)	0.169	1.28 (0.97–1.68)	0.083
Waist circumference, cm			0.099		0.147			0.073		0.221
< 88	15/136	1		1		19/137	1		1	
≥ 88	43/166	1.66 (0.89–3.10)		2.00 (0.79–5.09)		50/185	1.66 (0.94–2.95)		1.51 (0.77–2.97)	
Per 10 U	58/302	**1.28 (1.05–1.55)**	**0.013**	**1.41 (1.07–1.86)**	**0.016**	69/322	**1.18 (1. 01–1.38)**	**0.034**	**1.22 (1.01–1.46)**	**0.036**
Waist-to-hip ratio			0.058		0.318			0.104		0.301
≤ 0.85	9/103	1		1		15/108	1		1	
> 0.85	49/198	2.10 (0.97–4.52)		1.62 (0.60–4.39)		54/194	1.63 (0.89–3.00)		1.44 (0.71–2.91)	
Per 0.1 U	58/301	**1.74 (1.28–2.39)**	**0.001**	**1.92 (1.27–2.90)**	**0.002**	69/322	1.13 (0.86–1.48)	0.388	1.08 (0.77–1.53)	0.654
Metabolic syndrome (MetS)			**0.002**		**0.023**			0.885		0.890
No	18/164	1		1		36/193	1		1	
Yes	25/58	**2.84 (1.47–5.48)**		**2.81 (1.15–6.86)**		19/60	1.04 (0.58–1.89)		1.05 (0.50–2.23)	
MetS components			**0.036**	+	0.193			0.059		0.158
None	0/37					4/44	1		1	
None+1–2	15/135	1		1		30/121	**3.07 (1.07–8.81)**		2.91 (0.85–9.90)	
≥ 3	25/58	**2.98 (1.52–5.88)**	**0.002**	**2.80 (1.14–6.88)**	**0.03**	19/60	1.02 (0.56–1.86)	0.9	2.44 (0.66–9.04)	

^*^HR adjusted by terms of: age (≤ 40, 41–60, > 60), center (Pascale, Policlinico), Stage (I–IIA, IIB, IIIA–IIIC).

^**^For the entire variable the p-value refers to Wald Test, for numerical variable z-Test p-value was reported. +model was not implemented due to absence of events in reference category.

Significant results are shown in bold.

MetS components may not impact mortality to the same extent. Then, we investigated the impact of each MetS component on all-cause and BC-specific mortality across BMI categories (Additional Table 1).

Finally, we investigated the impact of adiposity through BMI categories in conjunction with MetS on BC-specific mortality (Additional Fig. 3).

## Discussion

Our data show that high BMI, central obesity and MetS are independently associated with an increased risk of all-cause and BC-specific mortality in BC survivors. The impact of anthropometric and metabolic parameters on long-term clinical outcomes varies depending on menopausal status and BC molecular subtype, with the most significant associations found in pre-menopausal patients and in women with luminal A-like malignancies. To the best of our knowledge, this is the first prospective study that evaluated the prognostic significance of anthropometric measurements and MetS components on mortality outcomes in a large cohort of BC survivors living in a Southern Mediterranean region.

In BC patients, obesity has been associated with more aggressive tumor characteristics, such as larger tumor size and higher grade, as well as with higher comorbidities^15^, reduced disease-free, overall and BC-specific survival^16–19^. Obesity and MetS could promote BC proliferation, invasion and progression through low chronic inflammation and imbalance of tumor microenvironment which result in increased production of fibroblasts, T cells and pro-inflammatory cytokines such as TNF-a, IL-6, and IL-8. Moreover, adipose tissue is associated with an increased production of the aromatase enzyme which promotes the conversion of androgens to estrogen. Furthermore, metabolic disorders cause an imbalance between increased production of leptin, considered a biomarker of the MetS and decreased secretion of adiponectin, an anti-inflammatory adipokine^20^.

A prognostic role of obesity, evaluated according to BMI categories, has been reported both at baseline and after BC diagnosis^21^. In our study, each 5.0-U increase in BMI increased all-cause and BC-specific mortality in the overall study population. However, the magnitude of the effect on survival mostly resulted from the prognostic impact of obesity among pre-menopausal women. High BMI is commonly used as a proxy of obesity because of easy accessibility of patient height and weight in retrospective studies, while anthropometric measures, such as WC and WHR, are not routinely collected in clinical practice. However, BMI may not fully capture or distinguish several anthropometric and metabolic alterations that are associated with obesity in cancer patients. In addition, BMI does not take into account absolute and relative lean body mass^22^. On the other hand, WC and WHR may better reflect body fat distribution and the presence of central obesity^12^. In a population of Black BC survivors, Bandera et al.^7^ found that high WC and WHR are associated with a significantly increased risk of death after BC diagnosis, with less stronger results for BMI^7^. In the present study, adiposity was evaluated using 3 measurement methods, namely BMI, WC and WHR. However, our data also confirms the relevance of central obesity on all-cause and BC-specific mortality. In detail, each 10-U increase in WC and every 0.1-U increase in WHR were associated with increased all-cause and BC-specific mortality in the overall study population, and particularly in pre-menopause. Together, these results suggest that central obesity may be especially detrimental in younger BC survivors, and that lifestyle interventions aimed at preventing or reversing central obesity are a clinical priority in these patients.

We previously showed that MetS is associated with an increased risk of BC recurrence and mortality^23^. Specifically, BC patients with 1-2 MetS components had a higher risk of all-cause and BC-mortality when compared to patients without MetS components^23^. Herein we confirm our earlier findings and we also show that the presence of even a single MetS component was associated with significantly higher all-cause and BC-specific mortality compared to patients without MetS components.These findings are consistent with the prospective investigation of Dibaba et al.^24^, where women with BC and MetS showed a 73% increased risk of BC-specific mortality at 14-year follow-up. Similar to our findings, the risk of BC-specific mortality increased as the number of MetS components increased and reached significance only in post-menopausal women with ≥ 3 MetS components, while no associations were found in pre-menopausal women^24^.

However, each MetS component may not impact survival to the same extent (Additional Table 3). We found that hypertriglyceridemia was the component affecting mortality the most including in women with normal BMIs. On the contrary, hyperglycemia could determine worse outcomes particularly in patients with obesity. Multiple studies confirmed these associations^25,26^. Taken together, these observations may be of particular clinical relevance because they suggest that a close monitoring of patient serum triglycerides or glucose concentrations, as well as prompt correction of dysregulated serum triglyceride and glucose levels through physical activity, lifestyle or pharmacologic interventions, may improve the prognosis of patients with surgically resected early BC.

There is evidence that the association between obesity or metabolic disorders and BC prognosis varies according to BC subtypes, with fairly consistent results for ER-positive BCs, but not for other BC subtypes^15^. We found that central obesity was associated with higher risks of all-cause and BC-specific mortality in HR+ BC, especially luminal A-like, while we found no clear associations in HER2+ and TNBC. Because obesity is associated with elevated aromatase activity and serum estrogen levels in post-menopausal women, it is possible that obesity modulates responses to endocrine therapy as shown in several studies^27,28^. In pre-menopausal patients, a similar pattern was seen in the Austrian Breast and Colorectal Cancer Study Group 12 trial, in which anastrozole plus goserelin was associated with higher risk of tumor recurrence and death in both overweight and obese women when compared with tamoxifen plus goserelin, whereas disease-free survival and overall survival were similar in the two treatment cohorts among women with normal-weight^29^. The influence of BMI on sex hormone levels was investigated in the Tamoxifen and Exemestane Trial (TEXT) and Suppression of Ovarian Function Trial (SOFT), which investigated exemestane versus tamoxifen plus ovarian suppression^30,31^. In these trials a higher BMI was associated with a higher likelihood of elevated estradiol during treatment^32^. In contrast, a recent meta-analysis reported that general obesity was associated to higher all-cause mortality regardless of molecular subtype^33^. Similar to our findings, a more recent meta-analysis showed that obesity was associated with all-cause and BC-specific mortality in HR+/HER2-, and HER2+ BC, while no clear associations were observed in TNBC^34^. In our study MetS was associated with all-cause and BC-specific mortality in HR+ and HER2+ BC and to a lesser extent in TNBC. Biologic factors involved in MetS, namely insulin resistance, hyperinsulinemia, hyperglycemia, altered adipokines and inflammation are potentially relevant across BC subtypes, regardless of endogenous estrogen levels^35^.

Our study has several strengths. Firstly, it centers on a large, high quality, multicenter cohort of BC survivors. The data were prospectively collected, and the clinical and tumor features were annotated and for whom complete information on MetS components, anthropometric indices and measurements, subsequent treatment and clinical outcomes are available. Main limitations of this study are the presence of missing values for those variables which are related with our outcome:

(a) limited information on existing comorbidities and concomitant therapies; (b)the absence of information on body weight and anthropometric measurements before and after diagnosis as well as during the follow-up period to investigate changes from baseline and their associations with survival outcomes; (c) although anthropometric measurements (body weight, WC, WHR) are low cost, easy-to-collect and to use in daily clinical practice, their use can be problematic due to their vulnerability to measurement errors and lack of reliability; (d) similarly, nutritional status and in particular body fat measurement was not evaluated using more accurate methods, for example Dual-Energy X-Ray Absorption or Bioelectrical Impedance Analysis; (e) it is possible that lifestyle habits including diet may differ among lean and obese individuals which we did not collect at baseline however our study cohort living in a Southern Mediterranean region of Italy is characterized by Mediterranean dietary traditions which did not change significantly in the last decade^36^. Nevertheless, the consistency between BC-specific mortality and all-cause mortality results are pressuring in this perspective.

In conclusion, our data confirm and expand previous data showing an association between central obesity and an increased risk of death. The magnitude of this effect (35 to 40% increased risk) suggests that obesity may impair the effectiveness of BC therapies. Based on our findings, future prospective trials should investigate if lifestyle changes, such as nutritional or physical activity interventions, which are capable of positively modifying anthropometric and metabolic parameters, are also associated with improved clinical outcomes. In this respect, the multicenter, randomized, phase III trial BWEL (NCT02750826) investigated if promoting weight loss interventions in surgically-resected, overweight or obese BC patients results in a reduction of BC recurrences. Results of this trial are highly expected.

### Supplementary Information

Supplementary Information.

## Data Availability

The data underlying this article are available in Zenodo at https://doi.org/10.5281/zenodo.8058949.

## Abbreviations

BC: Breast cancer
BMI: Body mass index
WC: Waist circumference
WHR: Waist-to-hip ratio
MetS: Metabolic syndrome
TNBC: Triple negative breast cancer
