# Supplementary material for: Central obesity, body mass index, metabolic syndrome and mortality in Mediterranean breast cancer patients
Source: Sci Rep. 2023 Dec 1;13:21208. doi: 10.1038/s41598-023-45439-y (PMC10692221; doi:10.1038/s41598-023-45439-y)
Supplement: Supplementary file 1 — Supplementary Information. [file 41598_2023_45439_MOESM1_ESM.docx]

**Supplementary figures**

**Additional Table 1.** Association of MetS components with breast cancer-specific mortality across BMI categories

|  | **Breast Cancer Specific mortality** | | | | | | | | |
| --- | --- | --- | --- | --- | --- | --- | --- | --- | --- |
| Variables | **BMI<25** | | | **BMI 25-29** | | | **BMI≥30** | | |
| ***Blood Pressure*** | N | HR^*^ (CI 95%) | p-value | N | HR^*^ (CI 95%) | p-value | N | HR^*^ (CI 95%) | p-value |
| *<130/<85mmHg* | 25/162 | 1 |  | 19/100 | 1 |  | 13/67 | 1 |  |
| *≥130/≥85mmHg* | 7/73 | 0.45 (0.18-1.12) | 0.087 | 13/98 | 0.79 (0.35-1.81) | 0.583 | 38/92 | **2.4 (1.17-4.95)** | **0.017** |
| ***HDL*** |  |  |  |  |  |  |  |  |  |
| *≥50mg/dL* | 22/143 | 1 |  | 14/119 | 1 |  | 26/80 | 1 |  |
| *<50mg/dL* | 8/31 | 1.92 (0.82-4.46) | 0.132 | 11/32 | 2.4 (0.97-5.93) | 0.059 | 9/42 | 0.51 (0.21-1.21) | 0.127 |
| ***Triglycerides*** |  |  |  |  |  |  |  |  |  |
| *<150mg/dL* | 24/236 | 1 |  | 21/177 | 1 |  | 29/137 | 1 |  |
| *≥150mg/dL* | 10/13 | **5.43 (2.29-12.89)** | **< 0.001** | 11/28 | **2.95 (1.2-7.26)** | **0.018** | 19/35 | **2.36 (1.25-4.45)** | **0.008** |
| ***Fasting Glucose*** |  |  |  |  |  |  |  |  |  |
| *<110mg/dL* | 27/240 | 1 |  | 31/182 | 1 |  | 29/138 | 1 |  |
| *≥110mg/dL* | 7/14 | 1.96 (0.72-5.3) | 0.187 | 5/34 | 0.98 (0.33-2.93) | 0.975 | 22/41 | **2.48 (1.36-4.53)** | **0.003** |
| ***Waist Circumference*** |  |  |  |  |  |  |  |  |  |
| *<88 cm* | 29/252 | 1 |  | 9/91 | 1 |  | 2/4 | 1 |  |
| *≥88 cm* | 6/21 | **2.71 (1.08-6.84)** | **0.034** | 25/150 | 1.79 (0.77-4.16) | 0.174 | 52/183 | 0.9 (0.2-4.05) | 0.887 |

^*^HR adjusted by terms of: age (≤40, 41-60, >60), Center (Pascale, Policlinico), Stage (I-IIA, IIB, IIIA-IIIC).

Hypertriglyceridemia (fasting, at least 8-hour fasting concentration of serum triglycerides ≥ 150 mg/dL) and higher WC (WC*≥*88cm) were associated with an increased risk of BC-specific mortality particularly in normal-weight patients ((BMI<25 kg/m^2^, HR=5.43, 95%CI: 2.29-12.89 and HR=2.71, 95% CI 1.0-6.84, respectively). Higher blood pressure (blood pressure ≥ 130/ ≥ 85 mmHg) and hyperglycemia (fasting plasma glucose concentration ≥ 110 mg/dL) were associated with a higher risk of BC-specific mortality in obese patients (BMI≥30 kg/m^2^ , HR=2.4, 95%CI: 1.17-4.95 and HR=2.48, 95%CI: 1.36-4.53, respectively)

**Additional Figure 1.** Overall and breast cancer-specific survival curves


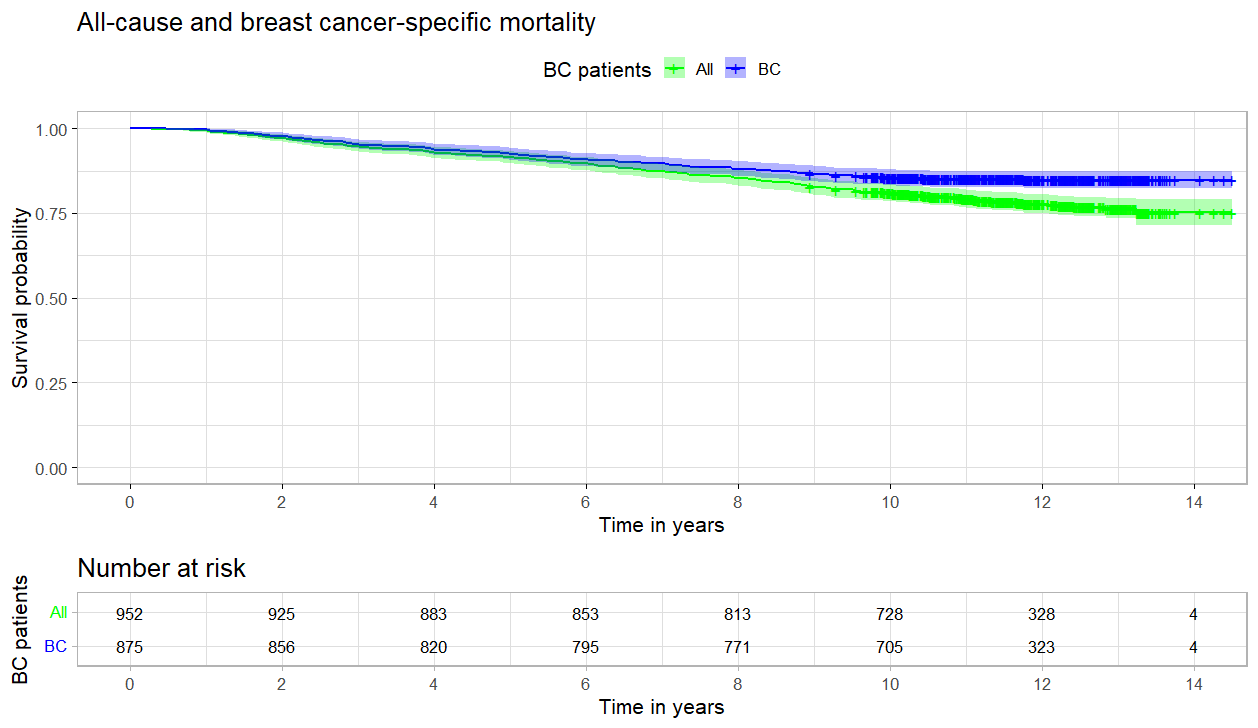


**Additional Figure 2.** All-cause (left) and breast cancer-specific (right) mortality by body mass index (BMI, kg/m^2^), waist circumference (WC, cm), waist to hip ratio (WHR), metabolic syndrome (MetS) and BC subtypes.


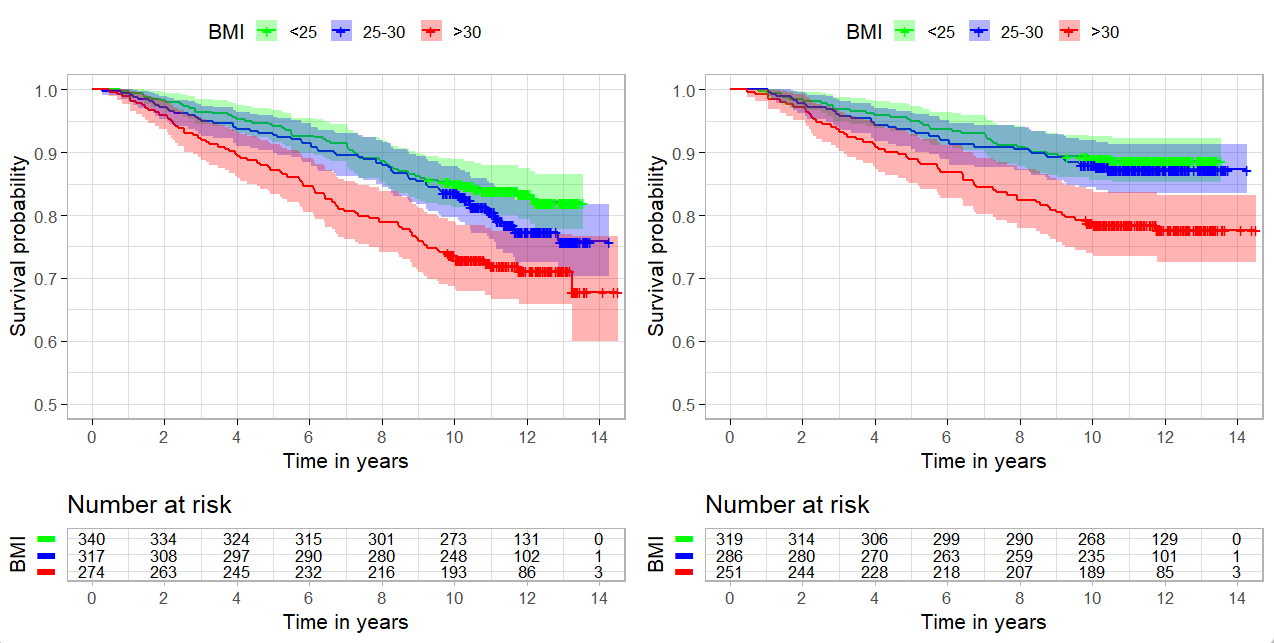


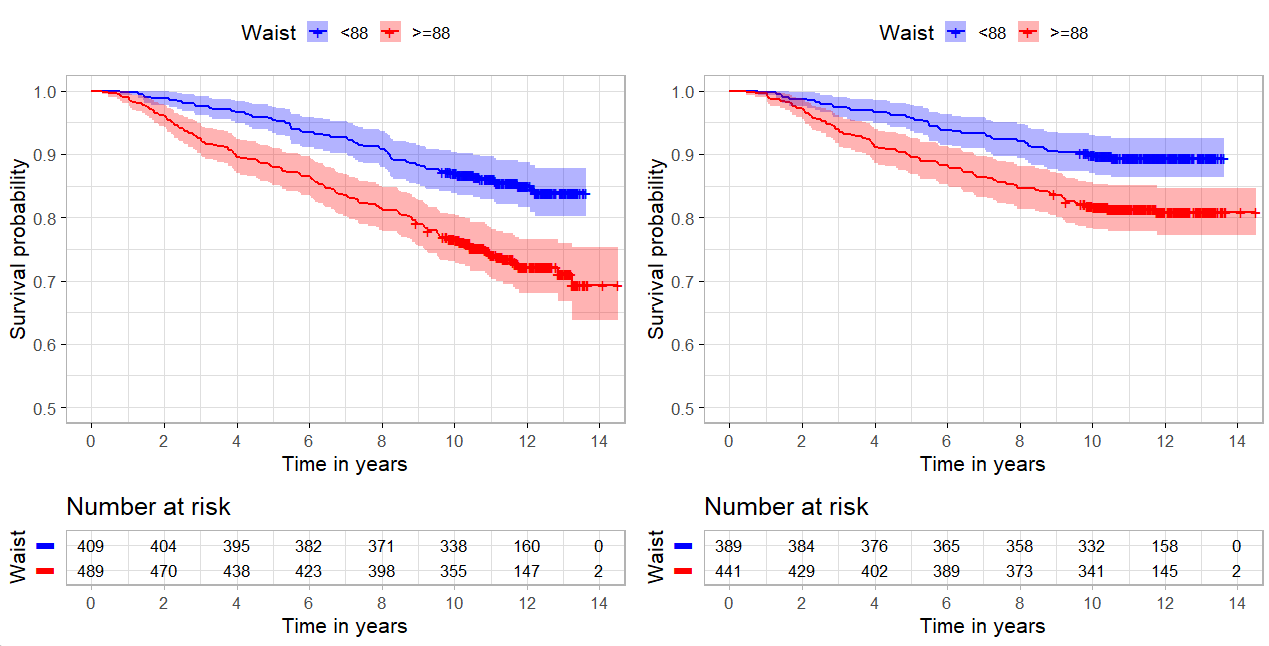


**
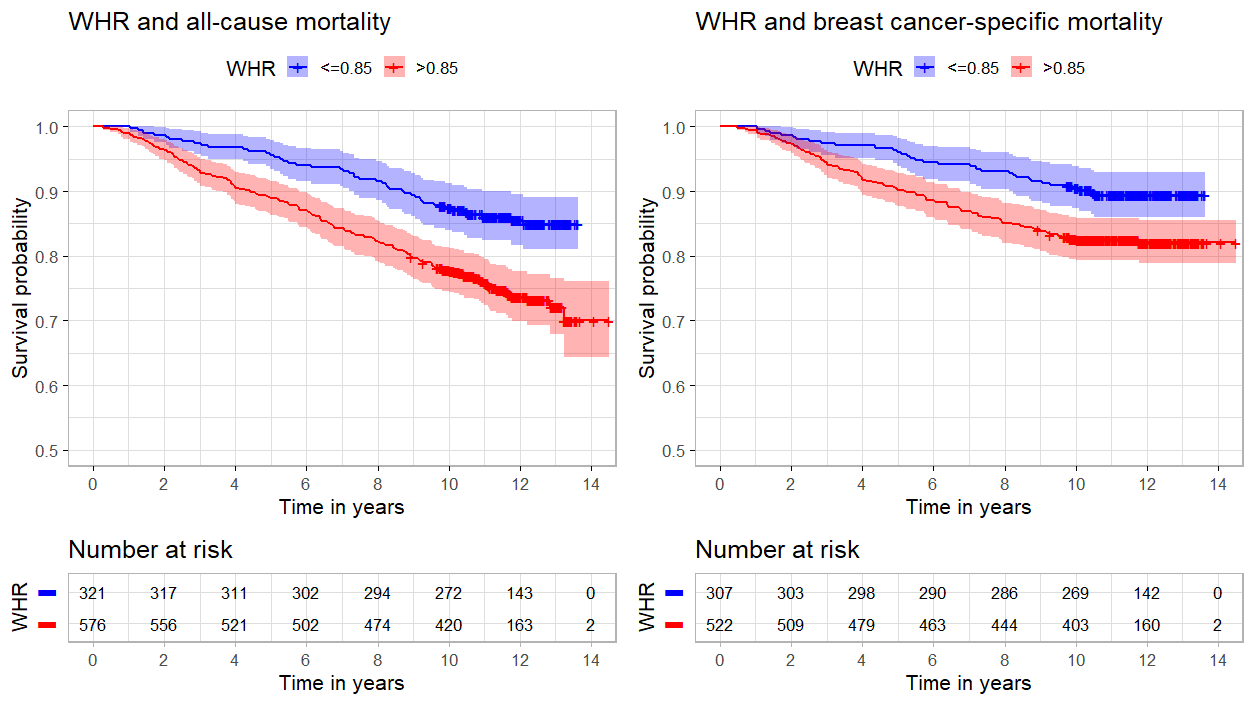
**

**
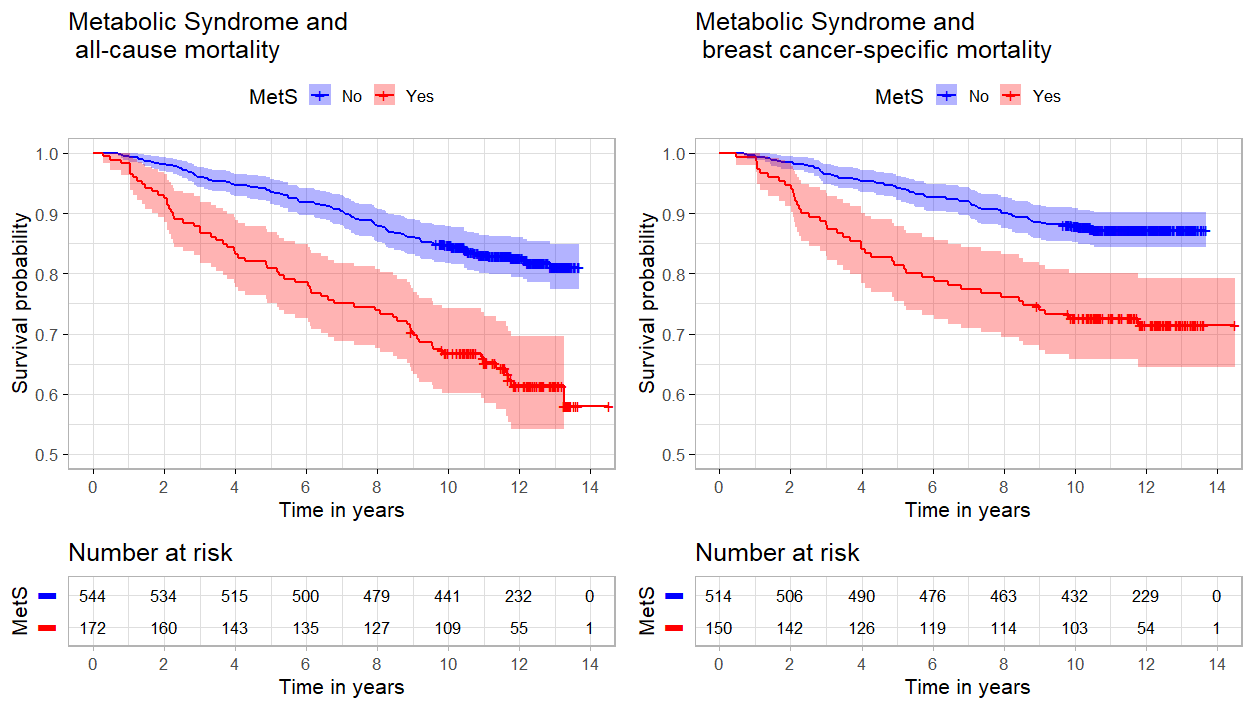
**

**
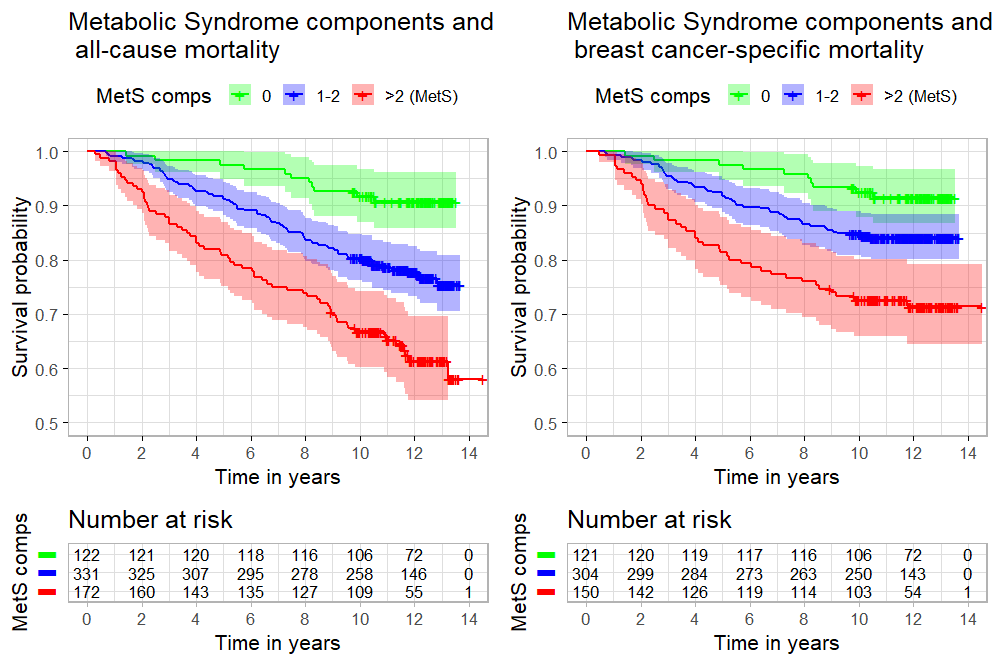
**

**
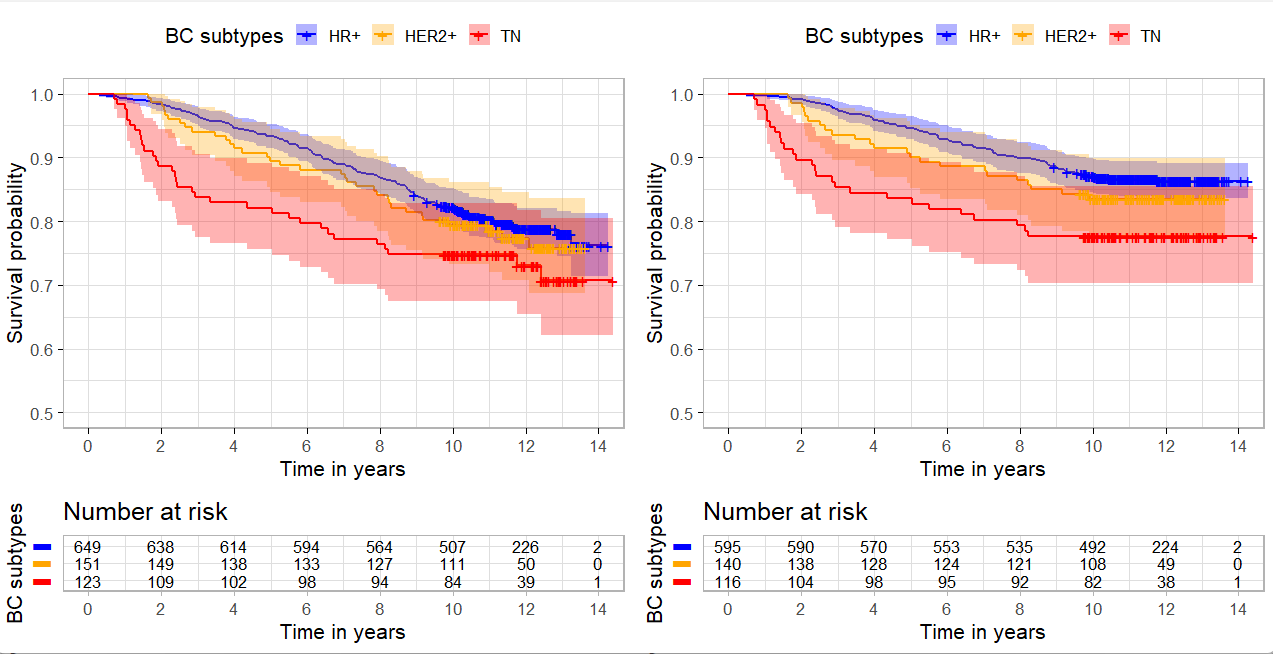
**

**Additional Figure 3.** Associations of adiposity across BMI categories in conjunction with Metabolic Syndrome (MetS) on BC-specific mortality


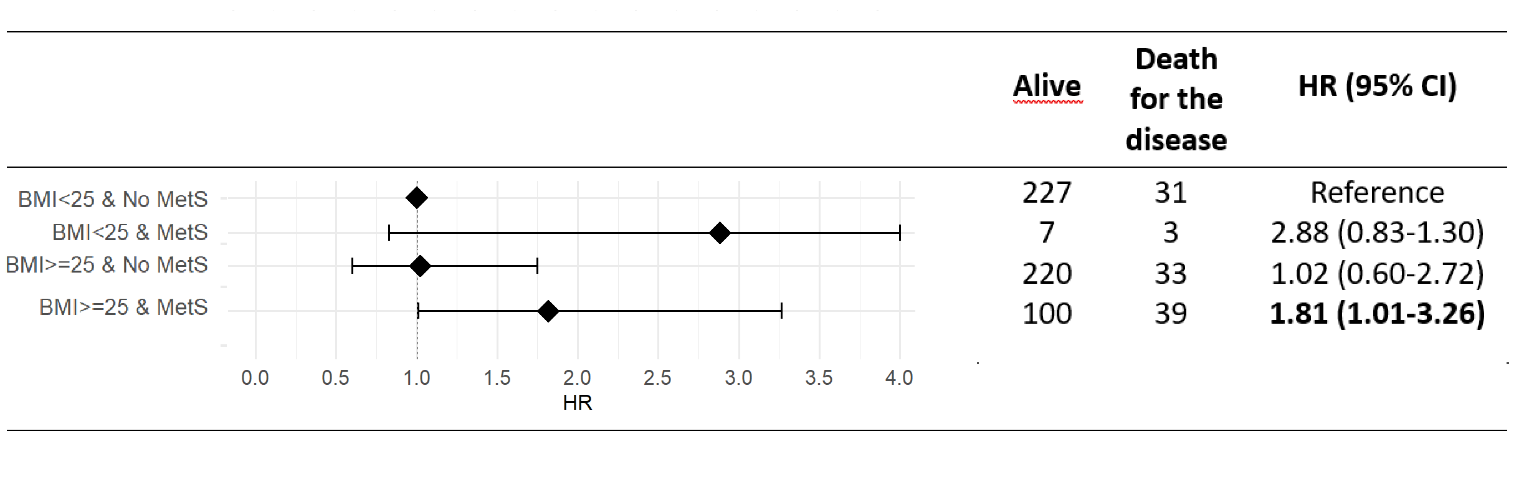


*Abbreviations: HR, hazard ratio; BMI, Body Mass Index (BMI is calculated as weight in kilograms divided by height in meters squared); MetS, Metabolic Syndrome (defined according to NCEP-ATP III criteria).*

Additional Figure 3 shows associations of adiposity across BMI categories in conjunction with Metabolic Syndrome (MetS) on BC-specific mortality. Patients with higher BMI (BMI≥25 kg/m^2^) in conjunction with MetS had an increased risk of BC-specific mortality (HR=1.81, 95%CI: 1.01-3.26)

This supplementary material has been provided by the authors to give readers additional information about their work.
